# OASL as a Diagnostic Marker for Influenza Infection Revealed by Integrative Bioinformatics Analysis With XGBoost

**DOI:** 10.3389/fbioe.2020.00729

**Published:** 2020-07-02

**Authors:** Yang Li, Hongjie Liu, Quan Xu, Rui Wu, Yi Zhang, Naizhe Li, Xiaozhou He, Mengjie Yang, Mifang Liang, Xuejun Ma

**Affiliations:** ^1^NHC Key Laboratory of Medical Virology and Viral Diseases, National Institute for Viral Disease Control and Prevention, Chinese Center for Disease Control and Prevention, Beijing, China; ^2^BGI Education Center, University of Chinese Academy of Sciences, Shenzhen, China; ^3^ChosenMed Technology (Beijing) Co., Ltd., Beijing, China; ^4^Department of Pathology, Peking University Third Hospital, School of Basic Medical Sciences, Peking University Health Science Center, Beijing, China; ^5^Center for Biosafety Mega-Science, Chinese Academy of Sciences, Wuhan, China

**Keywords:** influenza infection, host response, OASL, XGBoost, WGCNA

## Abstract

Host response biomarkers offer a promising alternative diagnostic solution for identifying acute respiratory infection (ARI) cases involving influenza infection. However, most of the published panels involve multiple genes, which is problematic in clinical settings because polymerase chain reaction (PCR)-based technology is the most widely used genomic technology in these settings, and it can only be used to measure a small number of targets. This study aimed to identify a single-gene biomarker with a high diagnostic accuracy by using integrated bioinformatics analysis with XGBoost. The gene expression profiles in dataset GSE68310 were used to construct a co-expression network using weighted correlation network analysis (WGCNA). Fourteen hub genes related to influenza infection (blue module) that were common to both the co-expression network and the protein–protein interaction network were identified. Thereafter, a single hub gene was selected using XGBoost, with feature selection conducted using recursive feature elimination with cross-validation (RFECV). The identified biomarker was oligoadenylate synthetases-like (OASL). The robustness of this biomarker was further examined using three external datasets. OASL expression profiling triggered by various infections was different enough to discriminate between influenza and non-influenza ARI infections. Thus, this study presented a workflow to identify a single-gene classifier across multiple datasets. Moreover, OASL was revealed as a biomarker that could identify influenza patients from among those with flu-like ARI. OASL has great potential for improving influenza diagnosis accuracy in ARI patients in the clinical setting.

## Introduction

Acute respiratory infection (ARI) is responsible for significant levels of morbidity and mortality worldwide related to infectious diseases. Viruses and bacteria are the main causes of ARI. Among the viruses, influenza virus kills more people than other viruses. It has been estimated that there were 250,000–500,000 additional deaths during the first 12 months of the global circulation of the 2009 pandemic H1N1 influenza A virus (Dawood et al., 2012). Better diagnostics for ARI (with or without influenza virus) are urgently needed in both inpatient and outpatient settings. However, discriminating between influenza and non-influenza flu-like illnesses on clinical grounds is often difficult, because these ARIs share similar clinical features (e.g., cough and fever).

Diagnostic methods for viral pathogens, such as culture, serodiagnosis, nucleic acid-based methods, and high-throughput sequencing, are important to guide disease management. When the presence of a viral pathogen is confirmed by these methods, this does not exclude a possible coinfection with bacteria, leading to antimicrobial prescriptions “just in case” (Tsalik et al., 2016). Moreover, as for most respiratory pathogens, the presence of influenza virus is sometimes unrelated to the presenting illness (Jansen et al., 2011). There is currently widespread interest in tests for virus detection in general and tests for “active” virus detection.

**FIGURE 1:**
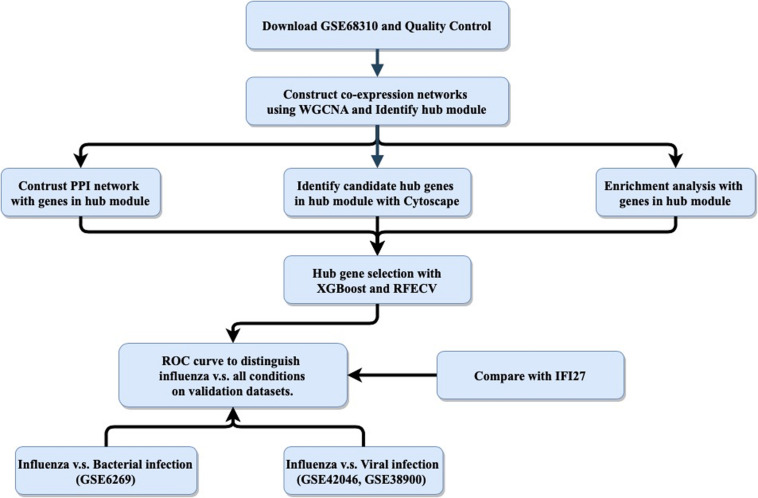
General study workflow: data collection, *in silico* analysis, and external validation. PPI, protein–protein interaction; RFECV, recursive feature elimination with cross-validation.

The host response to infection provides an alternative target for “active” virus detection. It has been reported that biomarkers based on host gene expression have great potential for distinguishing ARI patients infected with viruses versus bacteria (Herberg et al., 2016; Sweeney et al., 2016b; Tsalik et al., 2016; Yu et al., 2019). In addition to ARI, other infectious diseases such as tuberculosis (Sweeney et al., 2016a), systemic inflammation (Sampson et al., 2017) and hemorrhagic fevers (Robinson et al., 2019) have been studied using this approach. Most published panels for detecting the host response to infections contained multiple genes, making it difficult to apply them in clinical settings, as polymerase chain reaction (PCR)-based technologies could only measure a small number of targets. Recently, interferon alpha-inducible protein 27 (IFI27) was found to be able to distinguish influenza and non-influenza flu-like illnesses in a large cohort, with an area under the curve (AUC) value of 0.87 (Tang et al., 2017). However, IFI27 was the most upregulated gene during influenza virus, respiratory syncytial virus (RSV), and human rhinovirus (HRV) infections (Ioannidis et al., 2012; Zhai et al., 2015). Here, we aimed to follow the single-gene strategy to improve the discrimination between influenza and non-influenza flu-like illnesses based on an integrated bioinformatics analysis with XGBoost (Figure 1).

## Materials and Methods

### Study Design

The purpose of this study was to use an integrated bioinformatics analysis to analyze multiple gene expression datasets in order to identify a biomarker that can accurately classify patients with influenza or non-influenza flu-like illnesses, including bacterial infections and other viral infections. The general study workflow was shown in Figure 1.

### Data Collection

In brief, data were obtained from the Gene Expression Omnibus (GEO) database^1^ in December 2019 using the keyword “influenza cohort.” The following exclusion criteria were applied to the microarray data: (1) only involved influenza infection; (2) no or insufficient clinical data; (3) concerned influenza vaccine responses; and (4) used non-baseline (“healthy”) controls. After review, GSE68310, which contains 880 samples from 133 subjects with influenza infection or other viral ARIs, was selected for biomarker discovery (Zhai et al., 2015).

For the validation stage, three external independent microarray datasets were selected. GSE6269 (Ramilo et al., 2007) was used to evaluate the diagnostic performance between influenza and bacterial infections. Both GSE42026 (Herberg et al., 2013) and GSE38900 (Mejias et al., 2013) were used to estimate the discriminatory power to differentiate the influenza against other viral infections. In addition to controls, the three datasets contained cases with common bacterial and viral respiratory infections, i.e., *Streptococcus pneumoniae*, *Staphylococcus aureus*, influenza virus, HRV, and RSV etc. Before further analysis, the expression matrices were normalized and log2-transformed.

### Differentially Expressed Genes Screening

The limma R package was used to screen the influenza infection associated differential expressed genes (DEGs). DEGs analyses contrasting the Day 0 influenza A virus infected individual data with the baseline samples were performed by function for linear model fitting in the R package limma (Ritchie et al., 2015). Correction for multiple testing was addressed by controlling the false discovery rate (FDR) using the Benjamini–Hochberg (B.H.) method. Criteria for DEGs were an absolute log_2_ fold change (Log_2_FC) of 0 and the FDR-adjusted *P*-value of <0.05.

### Co-expression Network Construction

A co-expression network was constructed using the normalized GSE68310 data by the weighted correlation network analysis (WGCNA) in R (Langfelder and Horvath, 2008). Briefly, quality assessment of GSE68310 samples was conducted using the cluster method. The soft-thresholding power was then calculated, with the type of network set to signed. The correlation coefficient threshold was 0.90. Network construction was then performed based on the calculated power. In addition, the minimum number of genes in each module was 30 and the threshold for cut height was set to 0.25 to merge possible similar modules.

### Identification of Modules Related to Influenza Infection

For a given module, the expression profile was summarized into a single characteristic expression profile, designated module eigengenes (MEs). MEs were considered as the first principal component in the principal component analysis (PCA). Thereafter, a Pearson correlation analysis, calculating the Student asymptotic *P-*values for the correlations, between MEs and clinical traits (Progression, Baseline, Day0 of viral infection and gender) was conducted.

### Gene Ontology and Kyoto Encyclopedia of Genes and Genomes Analyses

To understand the functions of enriched genes in interesting modules, Gene Ontology (GO) (Ashburner et al., 2000) and Kyoto Encyclopedia of Genes and Genomes (KEGG) (Kanehisa et al., 2017) analyses were performed using clusterProfiler (Yu et al., 2012), identifying significant results based on a Benjamini–Hochberg FDR-adjusted *P*-value ≤0.05.

### Candidate Hub Gene Selection

Three bioinformatics approaches were combined to select the hub genes. First, the module that was most highly correlated with influenza infection was selected. Hub genes in the module were determined by both gene significance and module membership. Second, all the interesting genes were uploaded to the Search Tool for the Retrieval of Interacting Genes (STRING) database^2^ to create a protein–protein interaction network (PPIN) (Szklarczyk et al., 2019). Hub genes in PPIN were selected by maximum neighborhood component (MNC), degree and maximal clique centrality (MCC) using cytoHubba with Cytoscape (Shannon et al., 2003; Chin et al., 2014). Thereafter, hub genes common to both networks were chosen. Finally, a single hub gene was selected using XGBoost with recursive feature elimination with cross-validation (RFECV) (Pedregosa et al., 2011; Chen and Guestrin, 2016).

### External Dataset Validation of the Hub Gene

We validated the hub gene-based classification performance related to distinguishing influenza and non-influenza acute respiratory illness using the external datasets GSE6269, GSE42026, and GSE38900. We also compared the performance of the selected hub gene to the performance of IFI27, which is a biomarker that discriminates influenza from all other conditions, with an AUC value of 0.87 (Tang et al., 2017). Additionally, a receiver operating characteristic (ROC) curve was plotted, and AUC was calculated using “pROC” (Robin et al., 2011) to evaluate the performance of the selected hub gene regarding distinguishing influenza infection from all other conditions.

### Statistical Analysis

R (version 3.5.1) was used for most analyses, with hub gene selection being performed using XGBoost in Python (version 3.6). The statistical significance of pairwise differences between groups was analyzed using a two-tailed *t*-test. *P*-value ≤0.05 was considered statistically significant.

## Results

### Quality Control and Sample Selection

Raw data in dataset GSE68310 was subjected to background adjustment, variance stabilization after log2 transformation, rank invariant normalization, and quality control evaluation with a detection *P*-value less than 0.05 by using corresponding functions in the R package lumi (Du et al., 2008). The preprocessed expression matrix was then normalized by quantile method in R package limma. Thereafter, the probe sets with known gene symbol were kept, with 20,914 probes out of 47,254 remaining. No samples were removed after cluster analysis (Supplementary Figure S2).

### Influenza Associated DEGs

After quality control, we obtained the normalized expression matrices from GSE68310. Under the threshold of FDR < 0.05 and | log_2_FC| ≥ 0, a total of 6142 DEGs (2465 up-regulated and 3677 down-regulated) were achieved. The volcano plot of DEGs were shown in Supplementary Figure S2.

### Weighted Co-expression Network and Identification of the Influenza Infection-Related Module

**FIGURE 2:**
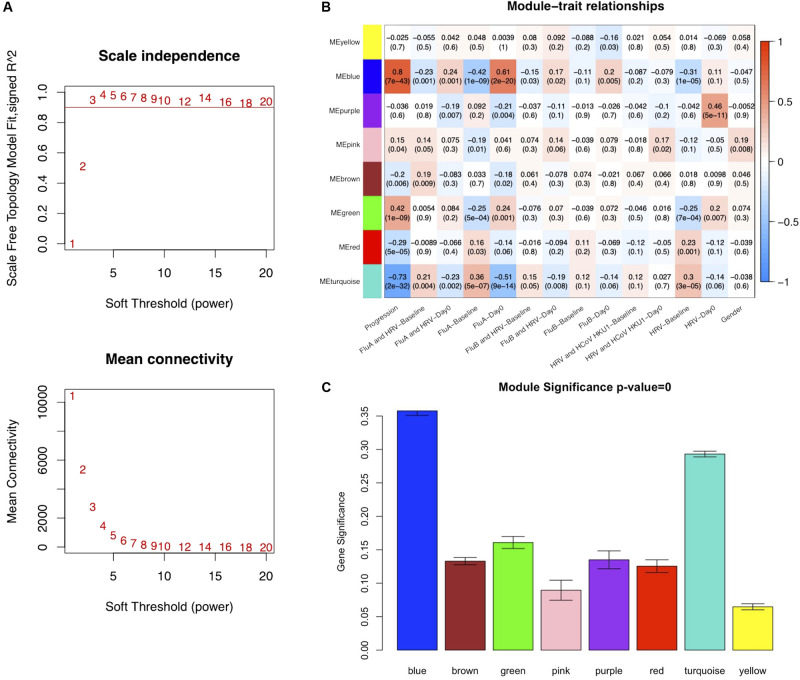
Co-expression network constructed using weighted correlation network analysis (WGCNA). **(A)** Analysis of the scale-free fit index with a threshold of 0.90 (top) and mean connectivity (bottom) for various soft-thresholding power values. **(B)** Distribution of average gene significance and errors in the modules associated with Influenza infections (FluA-Day0). **(C)** Heatmap of the correlation between module eigengenes and the clinical traits recorded in GSE68310. FluA, influenza A virus; FluB, influenza B virus; HRV, human rhinovirus; HCoV, human coronavirus.

To ensure that a scale-free network was constructed, a soft-thresholding power of 3 was selected while 0.90 was used as the correlation coefficient threshold (Figure 2A). After removing the gray module which contained unassigned genes (*n* = 10,047), a total of eight modules were identified and constructed in the WGCNA analysis (Figure 2B). The module with the most genes was the turquoise (*n* = 3127) module, followed by the blue (*n* = 1930), and brown (*n* = 1155) modules (Supplementary Figure S3). Modules with a greater MS were considered to have more connection with the influenza infections, and we found that the MS of the blue module was higher than those of any other modules (Figure 2C). In addition, module–trait correlation analyses showed that multiple modules were related to influenza infection. The Pearson correlation analysis, which involved calculating the Student asymptotic *P*-values for the correlations, between the MEs of each module and clinical traits is shown in Figure 2B. The blue module was the module most relevant to influenza infection, while the purple module was related to HRV infection.

### Quality Control of Modules Using Functional Analysis

**FIGURE 3:**
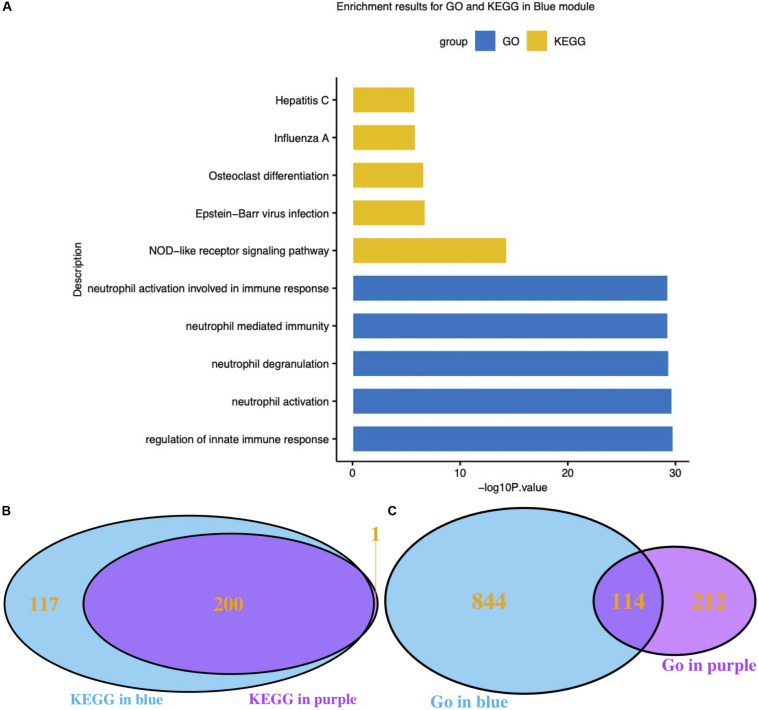
Functional analysis of interesting modules. **(A)** GO and KEGG enrichment results for the blue module; **(B)** Venn diagram of KEGG results for the blue and purple modules; **(C)** Venn diagram of GO results for the blue and purple modules. GO: Gene Ontology; KEGG: Kyoto Encyclopedia of Genes and Genomes.

Functional enrichment results of genes in the blue module, which was highly related to influenza infection, should hypothetically be related to the immune response to viruses. The GO and KEGG functional enrichment results were both used to examine this hypothesis (Figure 3A). The most highly enriched GO terms included regulation of innate immune response, neutrophil activation, neutrophil degranulation, neutrophil mediated immunity, and neutrophil activation involved in immune response. The KEGG results directly included the influenza A pathway (Figure 3).

It has been reported that different respiratory viruses can cause similar symptoms via different mechanisms. As the purple module was associated with HRV infection, GO and KEGG analyses were also performed on the genes in the purple module. The KEGG pathway results clearly suggested that the blue module (influenza-related) and the purple module (HRV-related) shared highly similar KEGG pathways (Figure 3B). Conversely, the GO Biological Process results were very dissimilar (Figure 3C). Thereafter, the correlation between module membership regarding the blue module and gene significance for HRV was assessed. No correlation was found, as shown in Supplementary Figure S4 (*r* = −0.11, *P* = 1.3e-6). Therefore, the presence of a unique set of genes in the blue module was correlated with influenza infections.

### Hub Gene Selection

**FIGURE 4:**
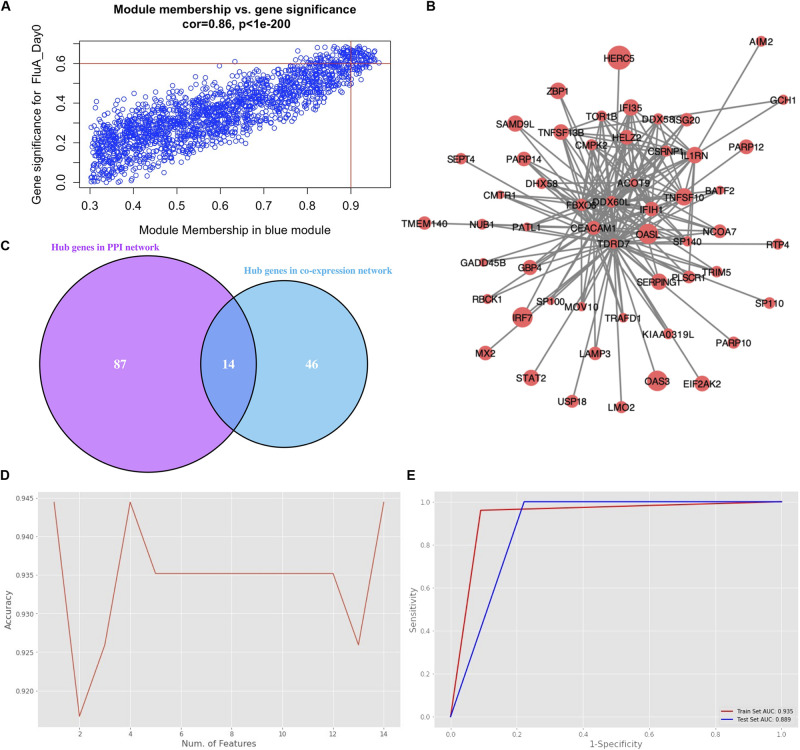
Hub gene selection. **(A)** Scatter plot of module eigengenes in the blue module with selection thresholds. **(B)** Visualization of the network connections among the most connected genes in the blue module. The size of circles was equal to the log_2_ fold change. **(C)** Common hub genes in both the PPI and co-expression networks. **(D)** Classification accuracy versus number of genes, based on the combination of XGBoost and recursive feature elimination with cross-validation. **(E)** Evaluation of classification performance of the selected hub gene, oligoadenylate synthetases-like (OASL), using dataset GSE68310.

The genes in the blue module were identified as candidate hub genes by the co-expression network approach. A total of 106 genes were selected using a gene significance threshold of 0.9 and a module membership significance of 0.6 (Figure 4A and Supplementary Table S1). In addition, the network connections among the most connected genes in the blue module was displayed through Cytoscape (Figure 4B). Next, a PPIN of all the genes in the blue module was constructed using Cytoscape based on the STRING database. The top 101 genes shared by MNC, degree and MCC through cytoHubba were considered as hub genes (Supplementary Table S1). Thereafter, 14 genes that were common to both networks were selected as the candidates to be further analyzed (Figure 4C and Supplementary Figure S5).

Hub gene selection based on XGBoost and RFECV was carried out using the 14 candidate genes. The samples labeled “Day0” (meaning that samples were collected within 48 h of ARI onset, i.e., in the acute phase) with data on the 14 genes were firstly standardized. They were then randomly assigned at a 7:3 ratio to a training set (93 samples) and a test set (40 samples). The “XGBoost” package in Python was used for data classification. Parameter max_depth was defined as 3; learning_rate was defined as 0.01; gamma was defined as 0.05; n_estimators was defined as 100. To obtain the best XGBoost model parameter combination (learning_rate, max_depth, gamma, and n_estimators) with the highest classification accuracy, fivefold cross-validation and grid search were applied to the training set. RFECV was then applied for feature selection based on the feature importance scores calculated by XGBoost. Parameter step was defined as 1; cv was defined as 5. The highest accuracy of classification was 0.944 which could be achieved through a single gene, oligoadenylate synthetases-like (OASL) (Figure 4D). Moreover, the AUC score in the training and test sets for this single gene was 0.935 and 0.889, respectively (Figure 4E).

### External Validation Cohorts

**FIGURE 5:**
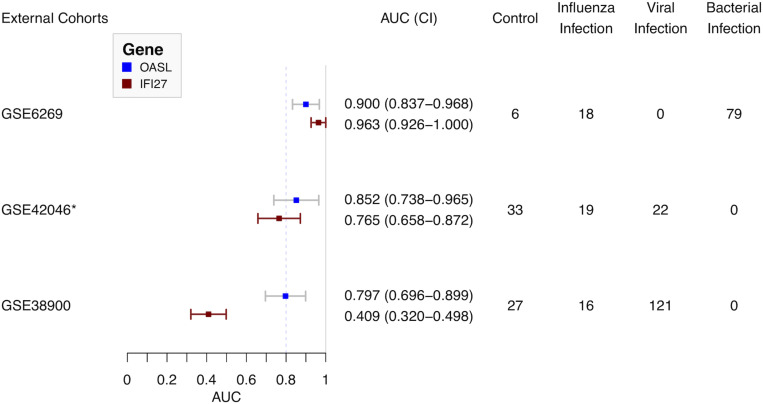
Forest plot of diagnostic performance of OASL and IFI27 on external cohorts. AUC, Area under curve. *Cases with bacterial infections were removed.

Three external cohorts were chosen to evaluate the diagnostic performance of the single gene-based classifier (Figure 5). First of all, GSE6269 was used to evaluate the diagnostic performance between influenza and bacterial infections. Both OASL and IFI27 showed high diagnostic accuracy (0.900 and 0.963, respectively). Next, GSE42026 and GSE38900 were used to estimate the discriminatory power to differentiate the influenza virus against other respiratory viruses. To meet this aim, cases with bacterial infection (*n* = 18) were firstly removed in GSE42026. After that, the AUC of OASL was 0.852 (95% CI: 0.738–0.965) while the AUC of IFI27 was 0.765 (95% CI: 0.658–0.872). For GSE38900, the AUC of OASL was 0.797 (95% CI: 0.696–0.899) while the AUC of IFI27 was 0.409 (95% CI: 0.320–0.498). AUC values were calculated using bootstrapping validation (Robin et al., 2011). Based on these findings, OASL achieved overall accurate results.

## Discussion

Over the last decade, considerable achievements have been made regarding the discovery of gene expression biomarkers of infections, especially respiratory illnesses (Herberg et al., 2016; Sweeney et al., 2016b; Tang et al., 2017; Robinson et al., 2019; Yu et al., 2019). In clinical settings, panels with multiple genes are problematic for infection diagnostics, as the most widely used genomic technology in clinical settings is PCR-based technologies, which can only be used to assess a handful of targets. To overcome this barrier, a single gene-based diagnostic strategy will be highly beneficial. IFI27 has recently been reported to be able to distinguish between influenza and bacterial infections (with an AUC of 0.91) and between influenza and non-influenza but flu-like illness (with an AUC of 0.87) (Tang et al., 2017). However, IFI27 has been found to be the highest upregulated gene during both influenza and RSV infections (Ioannidis et al., 2012). Therefore, an integrated bioinformatics analysis with machine learning was performed in this study to identify a hub gene that was specific to influenza infection.

As ARIs share similar clinical features and various respiratory viruses trigger a variety of interferon-stimulated genes (ISGs), an ideal dataset for biomarker discovery should include not only influenza infections, but also other respiratory infections. GSE68310 was finally selected (Zhai et al., 2015). To discriminate influenza infections from other viral infections, WGCNA, an unsupervised analysis method that clusters genes based on their expression profiles, was the first step to identify the hub module associated with influenza infection. Moreover, quality control involving enrichment analysis was performed on both the blue (influenza-related) module and the purple (HRV-related) module. Although diverse GO results were observed, similar KEGG pathways were enriched, which provides insights as to why the clinical features are similar among various viral infections (Figures 3B,C). The ISGs related to different viral infections were unique, which was consistent with previous research (Ioannidis et al., 2012; Andres-Terre et al., 2015). Therefore, the presence of a distinctive set of genes in the blue module was as expected.

To obtain a single hub gene for influenza infection, XGBoost was applied to the high-dimensional gene expression matrix. Compared with other ensemble machine learning algorithms, XGBoost extends simple classification and regression trees (CARTs) instead of building a single tree. Building many trees and then aggregating them to form a single consensus prediction model can improve the prediction accuracy (Chen and Guestrin, 2016). In addition, as a tree-based algorithm, XGBoost provided an importance score for each gene in each tree model. The importance score revealed how informative the gene was. RFECV showed good performance regarding feature reduction. Finally, the hub gene OASL was selected and tested in the discovery dataset GSE68310 (Figure 4).

To evaluate the diagnostic performance of OASL, three external datasets were selected (Figure 1). Firstly, both OASL and IFI27 shared similar highly accurate performance in discriminating between influenza and bacterial infections on GSE6269. To classify influenza and viral infections, OASL outperformed IFI27 slightly on GSE42026 with an AUC of 0.852 (95% CI 0.738–0.965) versus 0.765 (95% CI 0.658–0.872). In addition, we investigated another external cohort GSE38900 as a challenge dataset which contained 121 cases with non-influenza viral infections. Although both OASL and IFI27 showed reduced AUC on GSE38900, it was worth of noting that the AUC of OASL still remained close to 0.8. To avoid poor reproducibility across external patient populations, more studies with larger sample sizes were needed to verify the diagnostic performance of OASL.

Oligoadenylate synthetases-like, a member of the OAS family, mediates antiviral activities via promoting retinoic acid-inducible gene I (RIG-I)-mediated signaling by mimicking polyubiquitin (pUb) (Zhu et al., 2014). Notably, to evade host innate immunity, a number of viruses (especially influenza virus) target ubiquitin ligases or encode deubiquitinases (DUBs) and DUB-like molecules (Gack et al., 2009). Thus, in the absence of pUb (which is caused by influenza viruses), the activation of RIG-I triggered by OASL plays central roles in host antiviral activities. Recently, OASL has been considered as a new player in controlling antiviral innate immunity (Zhu et al., 2015). In addition, OASL was included by previous panels for discriminating viral and bacterial infections (Andres-Terre et al., 2015; Sampson et al., 2017). It was consistent with present results. OASL has considerable discriminatory power in differentiating between viral and bacterial infections (Figure 5). It was worthy of noting the expressions of OASL triggered by various viruses were different enough to tell influenza infection apart from other viral infections (Figure 5 and Supplementary Figure S8). The role of expressions of OASL triggered by different viruses in the pathogenesis of ARI need to be studied in the future.

Compared with other genomic technologies, influenza-targeted quantitative reverse transcription polymerase chain reaction (qRT-PCR) was widespread in clinical practice. The performance of PCR was limited because samples tend to be collected prior to ARI onset (and, sometimes, late in the illness), there is often a limited specimen quantity, and the nucleic acid (typically RNA) is often degraded. However, OASL was found to be upregulated during the progression of influenza infection (Supplementary Figure S9). To our surprise, OASL remained upregulated at 21 days after ARI onset which was the timepoint the subject had clinically recovered. The same trend was observed for IFI27 (Supplementary Figure S9). This might be caused by the influenza virus load was reduced but not eliminated. Therefore, identification of OASL expression might indicate the presence of an influenza infection when PCR indicated a negative result. As the OASL expression value was important and influenza is an RNA virus, we suggested using qRT-PCR to detect both OASL expression and influenza virus to distinguish between influenza and non-influenza flu-like cases in clinical settings.

Nevertheless, our study had certain limitations. First of all, the performances of OASL in the external datasets were moderate (AUC < 0.9). Secondly, limited types of viral infections were validated in the datasets. ARI is not caused by one or two viruses but a diverse viral community in the respiratory tract. We previously found that RSV, human coronaviruses (HCoV), human bocavirus (HBoV), influenza virus, human adenoviruses (HAdV), and human parainfluenza virus (HPIV) may be the main causes of severe ARI in Beijing, China (Wang et al., 2016). Thirdly, although it is accepted that the current study provides useful baseline data for future study, an ideal approach should be to perform a prospective study to verify the usefulness of OASL as an influenza ARI biomarker. Yet, it will be challenging to collect ARI specimens currently during the COVID-19 pandemic. Moreover, qRT-PCR is a commonly used validation tool for confirming gene expression results obtained from microarray. Therefore, we shall apply qRT-PCR to test the OASL assay’s accuracy with various ARI in the future work.

On the whole, this study addressed a major challenge related to translating genomic science into clinical practice. It has recently been reported that transcriptomes in nasal and blood samples from ARI patients exhibit similar patterns of type I interferon response (Yu et al., 2019). Thereafter, we suggested that a combination of both OASL and universal influenza detection, as measured by qRT-PCR using nasal samples, could be utilized to identify influenza infection in individuals with flu-like illness. Ultimately, before the OASL and influenza assay is used in clinical practice, there will be a need for prospective studies to establish its clinical utility as well as cost-effectiveness analyses.

## Data Availability Statement

The microarray datasets GSE68310, GSE6269, GSE42026, and GSE38900 for this study can be found in the Gene Expression Omnibus (GEO) database hosted by the National Center for Biotechnology Information of the US National Institutes of Health (https://www.ncbi.nlm.nih.gov/geo/).

## Author Contributions

YL, ML, and XM conceived of the study. YL, HL, and QX collected and analyzed the data. RW, YZ, NL, XH, and MY analyzed the data partially. YL and HL drafted the manuscript. ML and XM revised the manuscript. All authors read and approved the manuscript.

## Conflict of Interest

QX was employed by ChosenMed Technology (Beijing) Co. Ltd.

The remaining authors declare that the research was conducted in the absence of any commercial or OASL discriminated influenza infection financial relationships that could be construed as a potential conflict of interest.

## Footnotes

1 http://www.ncbi.nlm.nih.gov/geo/

2 https://string-db.org
