# Supplementary material for: OASL as a Diagnostic Marker for Influenza Infection Revealed by Integrative Bioinformatics Analysis With XGBoost
Source: Front Bioeng Biotechnol. 2020 Jul 2;8:729. doi: 10.3389/fbioe.2020.00729 (PMC7343705; doi:10.3389/fbioe.2020.00729)
Supplement: Supplementary file 1 [file Data_Sheet_1.docx]

OASL as a diagnostic marker for influenza infection revealed by integrative bioinformatics analysis with XGBoost


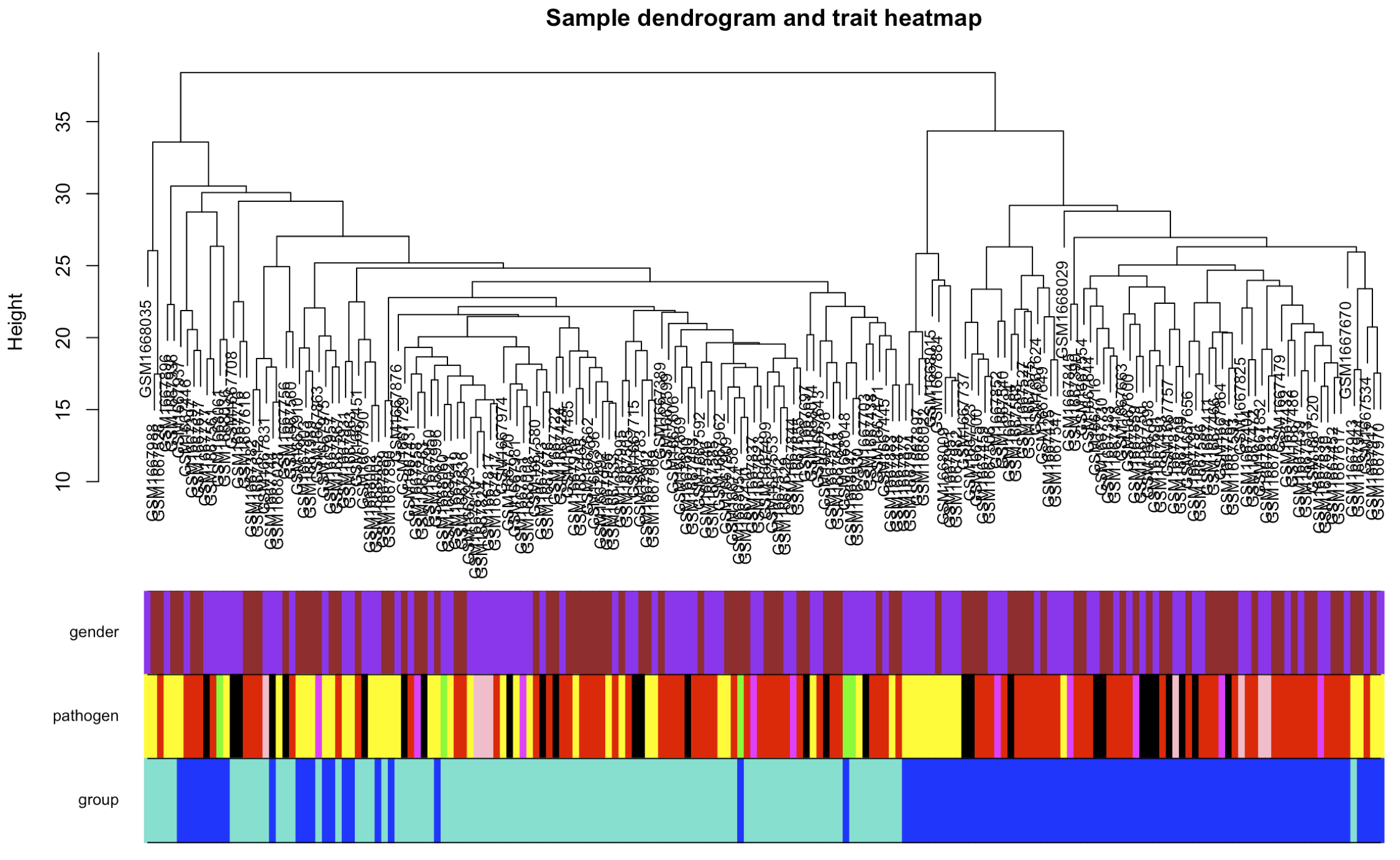


Figure S1 Samples clustering to detect outliers (GSE68310): sample dendrogram and trait indicator.


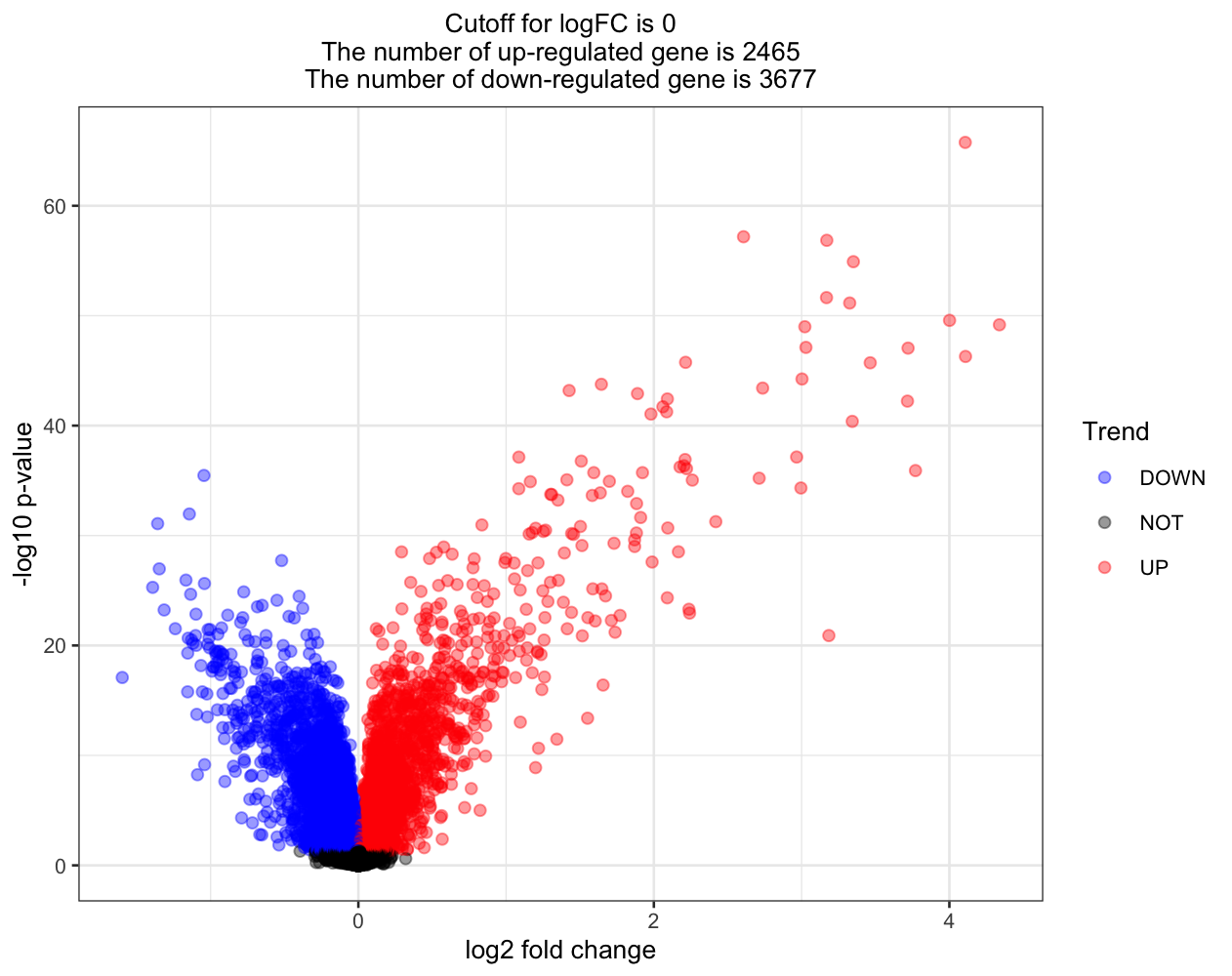


Figure S2 Volcano Plot of differentially expressed genes (DEGs) for influenza infection contrasting Day0 with baseline in GSE68310.


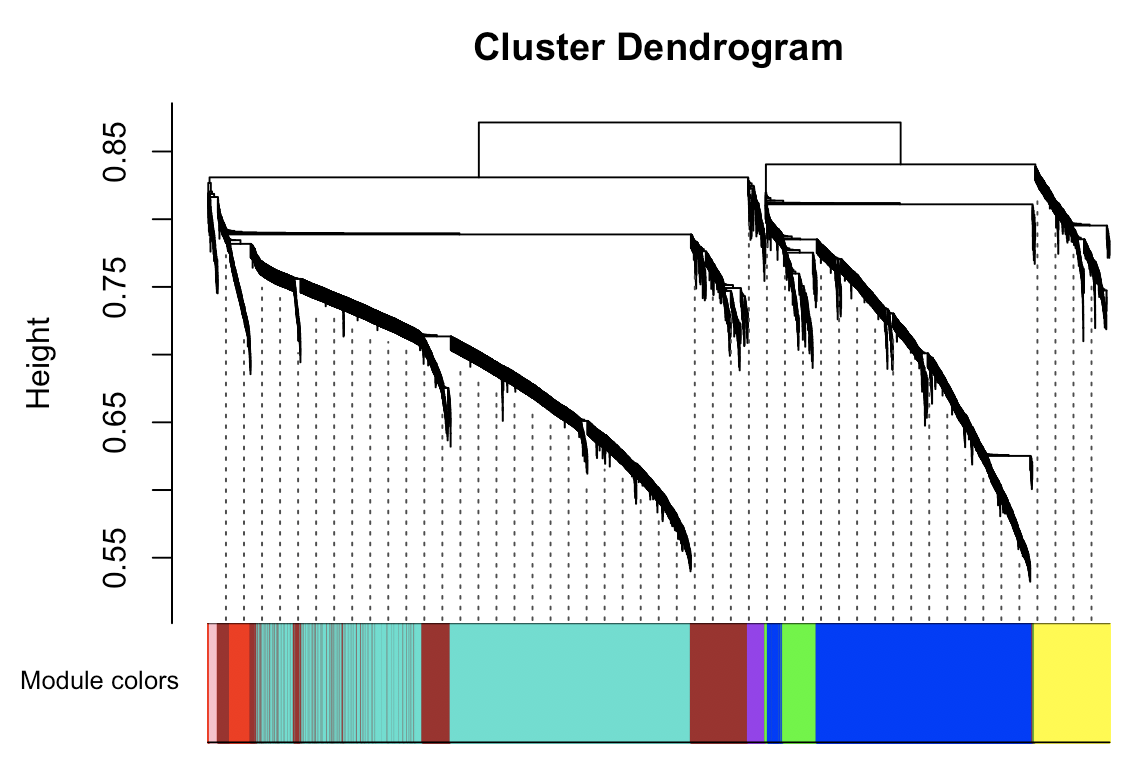


Figure S3 Dendrogram of all genes clustered based on a dissimilarity measure. The unassigned genes (grey module) were discarded.

Figure S4 Scatter plot of module eigengenes in blue module with trait HRV infection.


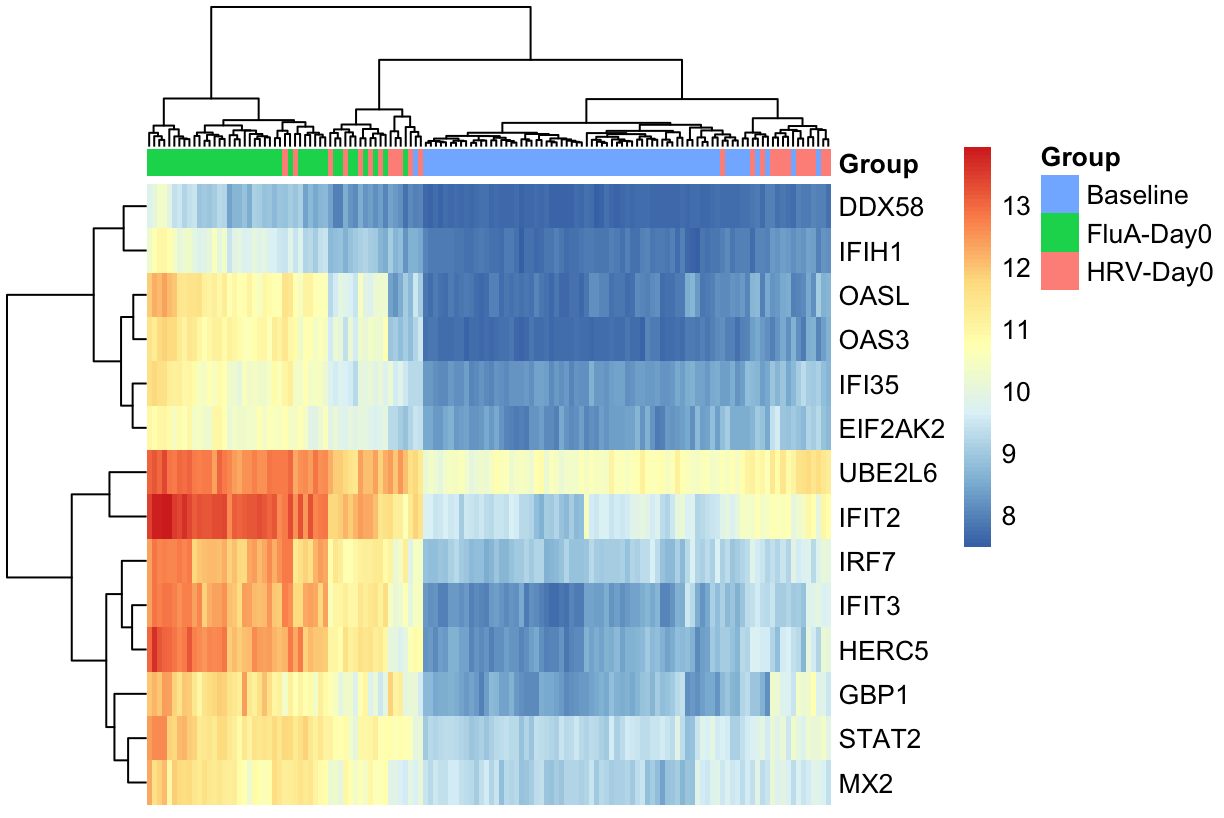


Figure S5 Heatmap based on unsupervised clustering of the selected 14 genes for influenza A virus (FluA), human rhinovirus (HRV) and baseline (controls). Each row represented one gene; each column represents one patient. Expression intensity is indicated by color (increased abundance in red, decreased in blue, intermediate in yellow).


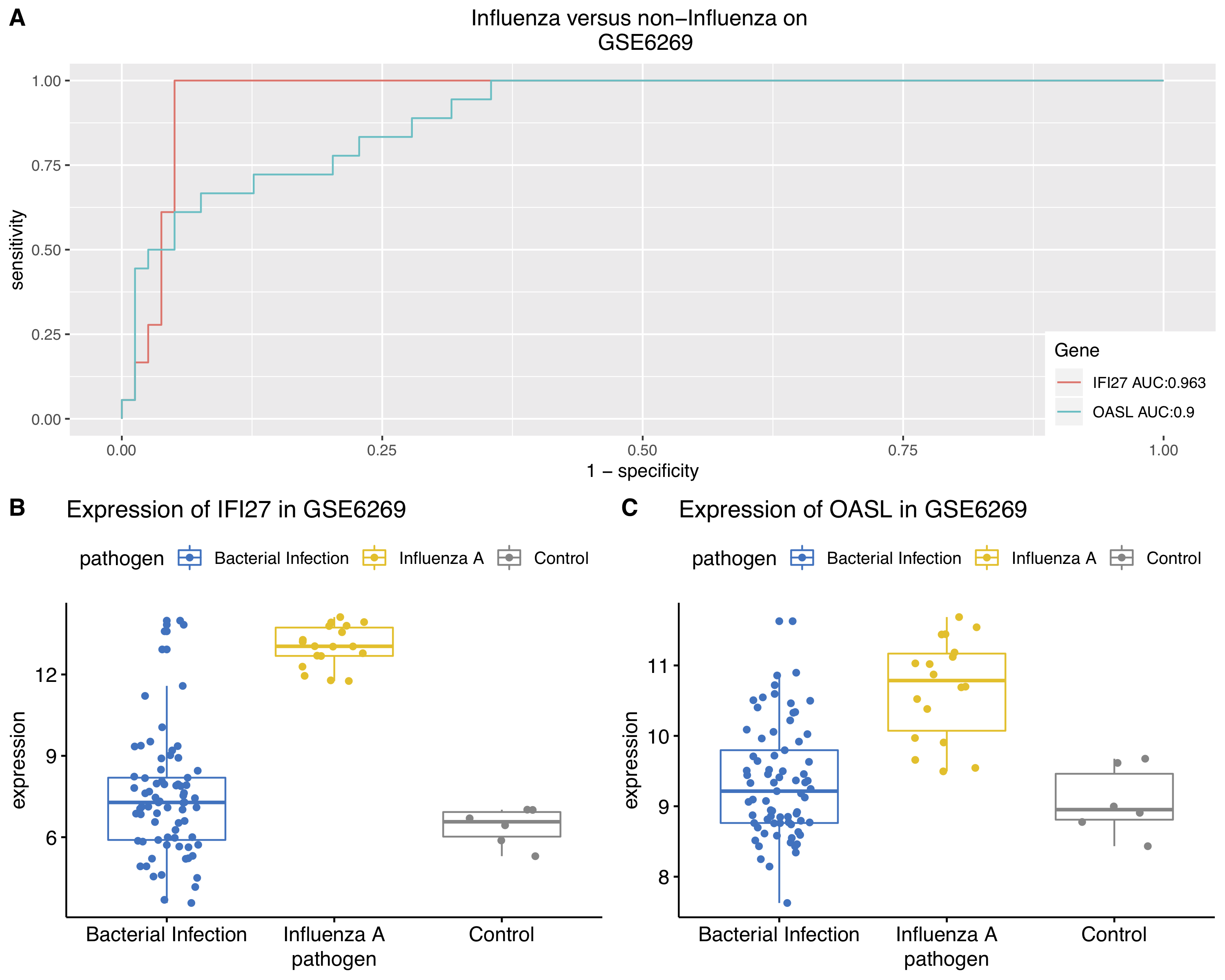


Figure S6 Comparison between OASL and IFI27 on GSE6269. (A) Diagnostic performance of OASL and IFI27 on GSE6269. (B) Expression of IFI27 in GSE6269. (C) Expression of OASL in GSE6269.


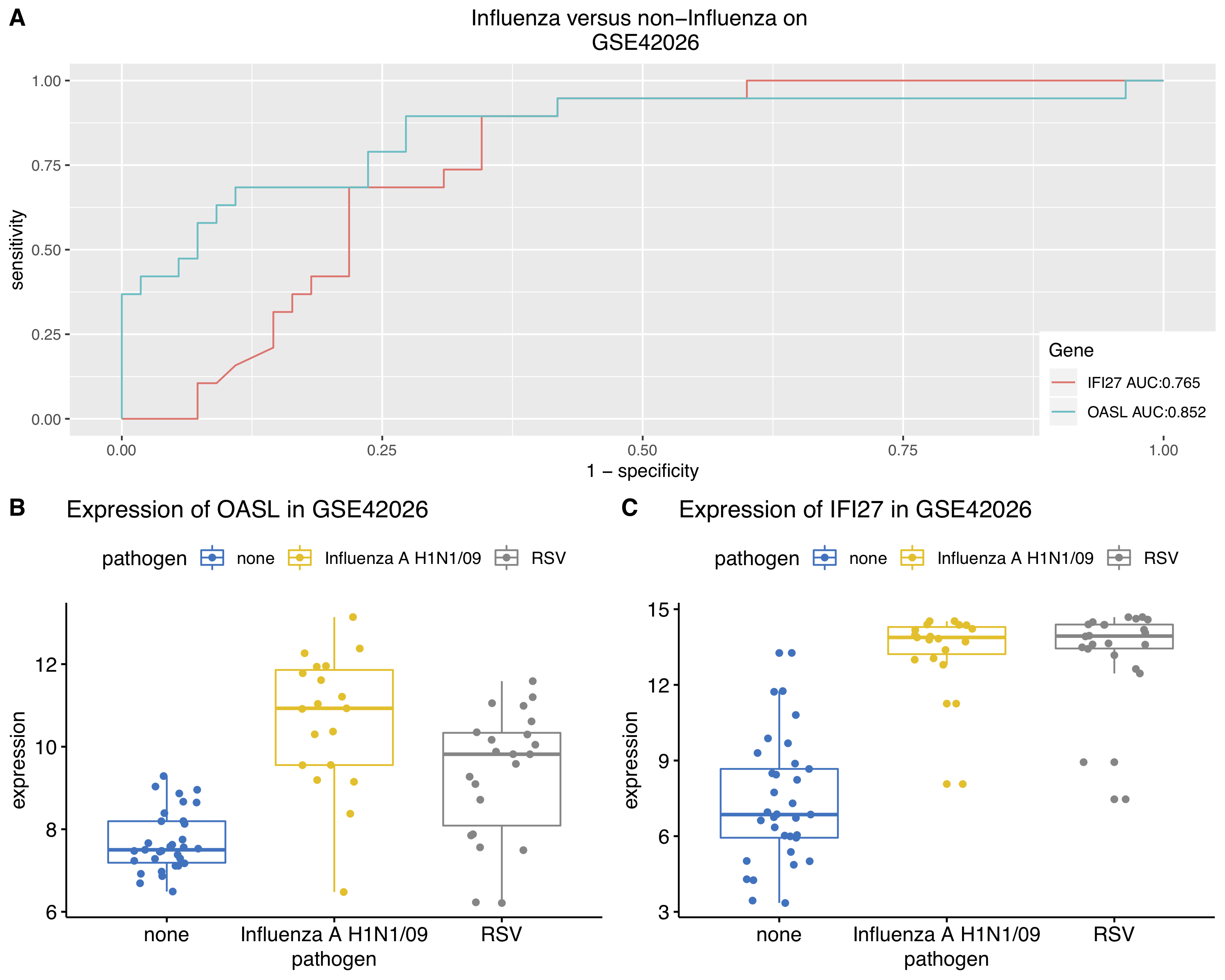


Figure S7 Comparison between OASL and IFI27 on GSE42026. (A) Diagnostic performance of OASL and IFI27 on GSE42026. (B) Expression of IFI27 in GSE42026. (C) Expression of OASL in GSE42026.


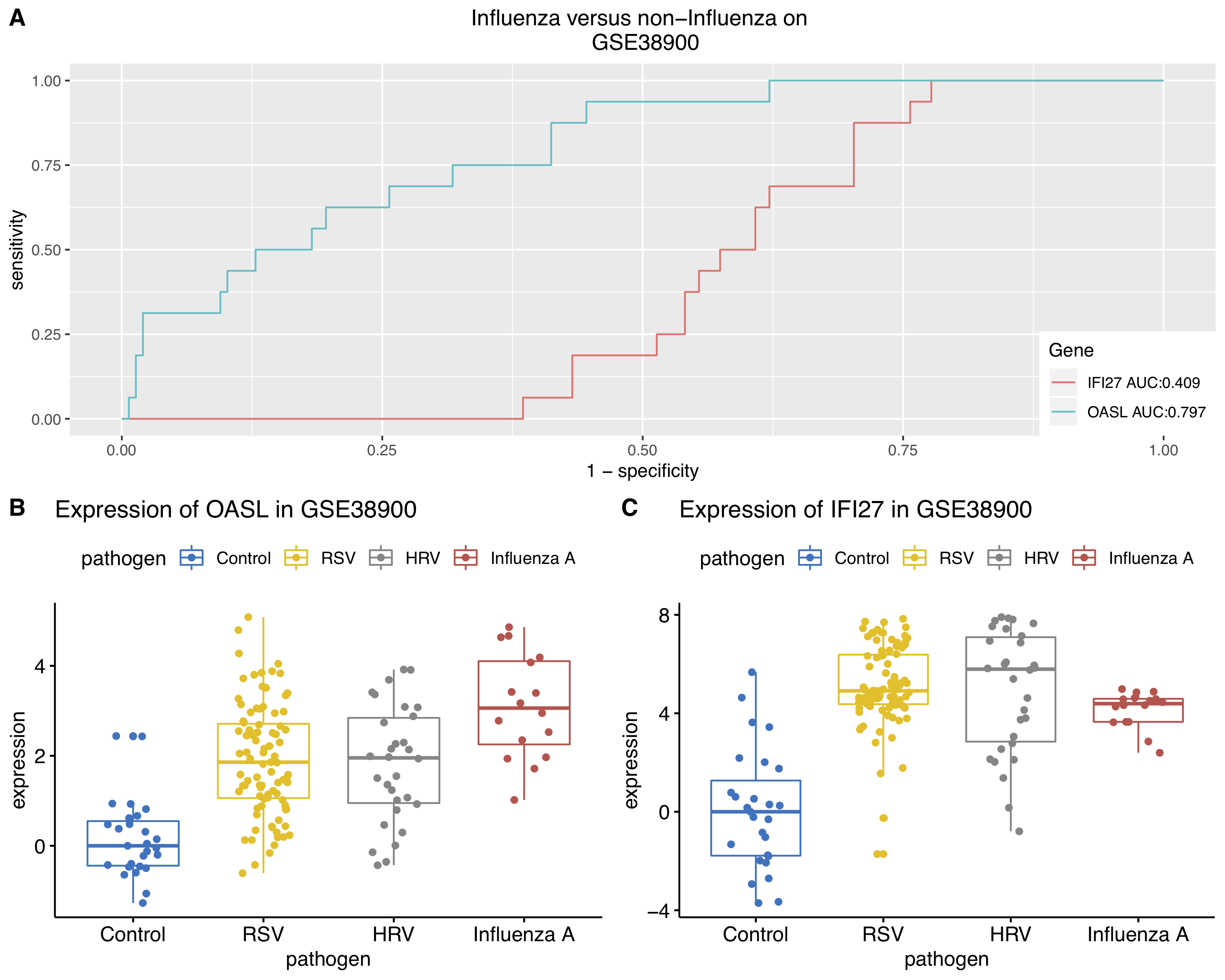


Figure S8 Comparison between OASL and IFI27 on GSE38900. (A) Diagnostic performance of OASL and IFI27 on GSE38900. (B) Expression of IFI27 in GSE38900. (C) Expression of OASL in GSE38900.

Figure S9 Expression of OASL and IFI27 along with progression of influenza infection in GSE68310. The reference group is baseline. Baseline, control samples; Day0, samples collected within 48 hours of ARI onset. ns: P > 0.05; *: P <= 0.05; **: P <= 0.01; ***: P <= 0.001; ****: P <= 0.0001.

Table S1 Candidate hub genes in blue module selected from co-expression network and protein–protein interaction network

| Selection approach | Candidate Hub Genes |
| --- | --- |
| Co-expression network (n = 60) | ACOT9, SERPING1, OASL, WDFY1, DHX58, PATL1, AIM2, LAP3, NCOA7, BATF2, STAT2, PARP14, IFIT5, FBXO6, IFIT3, CSRNP1, TRIM5, TDRD7, EIF2AK2, MICB, PANK2, IFI16, CEACAM1, TOR1B, HERC5, PARP9, SP110, TMEM140, IFIT2, PLSCR1, IFI35, OAS3, TAP1, SAT1, UBE2L6, GBP4, IL1RN, SEPT4, SP140, TRIM22, IFIH1, CMPK2, HELZ2, DDX60, DDX58, IRF7, SAMD9L, LMO2, TNFSF10, TMEM268, NTNG2, GCH1, SAMD9, TNFSF13B, GBP1, RTP4, MX2, NT5C3A, DDX60L, CMTR1 |
| Protein–protein interaction network (n = 101) | TNF, TLR4, STAT1, TLR2, STAT3, SRC, TLR8, IL1B, IRF7, TLR7, CXCL10, IRF1, CD86, MYD88, CASP3, SPI1, CREBBP, DDX58, TYROBP, MAPK14, IFIH1, ISG15, RELA, TRAF6, CCL2, MX1, FOS, PTEN, CASP1, C3AR1, B2M, CYBB, CYCS, SOCS3, PLEK, IRF9, CD40, TLR6, OASL, LILRB2, FCGR1A, TLR5, CUL1, BST2, IRF5, TRIM21, PSMB8, BRCA1, SOCS1, NOD2, CASP8, HLA-E, IFIT1, OAS1, HLA-A, JAK2, NLRP3, OAS2, RSAD2, HLA-C, STAT2, FPR2, HLA-B, TNFRSF1A, LCP2, UBE2L6, LYN, GBP1, HCK, MNDA, IFIT3, MX2, SELL, CD68, HIST2H2BE, TRIM25, DECR1, ATG7, IRF2, GRB2, TBK1, STAT5A, HLA-G, EGR1, HERC5, IFIT2, OAS3, FCER1G, RAB5A, HERC6, IFI35, BTK, FCGR2A, HIST2H2AC, GBP2, CD274, REL, UBE2D3, EIF2AK2, FCGR2B, ANXA5 |

| Gene  Table S2 Top 10 Functional Partners of OASL | Description | Score |
| --- | --- | --- |
| MX1 | Interferon-induced GTP-binding protein Mx1; Interferon-induced dynamin-like GTPase with antiviral activity against a wide range of RNA viruses and some DNA viruses. | 0.999 |
| IFIT1 | Interferon-induced protein with tetratricopeptide repeats 1; Interferon-induced antiviral RNA-binding protein that specifically binds single-stranded RNA bearing a 5’-triphosphate group (PPP-RNA), thereby acting as a sensor of viral single- stranded RNAs and inhibiting expression of viral messenger RNAs. | 0.997 |
| MX2 | Interferon-induced GTP-binding protein Mx2; Interferon-induced dynamin-like GTPase with potent antiviral activity against human immunodeficiency virus type 1 (HIV-1). | 0.997 |
| IRF7 | Interferon regulatory factor 7; Key transcriptional regulator of type I interferon (IFN)-dependent immune responses and plays a critical role in the innate immune response against DNA and RNA viruses. | 0.996 |
| IFIT3 | Interferon-induced protein with tetratricopeptide repeats 3; IFN-induced antiviral protein which acts as an inhibitor of cellular as well as viral processes, cell migration, proliferation, signaling, and viral replication. | 0.995 |
| ISG15 | Ubiquitin-like protein ISG15; Ubiquitin-like protein which plays a key role in the innate immune response to viral infection either via its conjugation to a target protein (ISGylation) or via its action as a free or unconjugated protein. | 0.994 |
| IFIT2 | Interferon-induced protein with tetratricopeptide repeats 2; IFN-induced antiviral protein which inhibits expression of viral messenger RNAs lacking 2’-O-methylation of the 5’ cap. | 0.993 |
| RSAD2 | Radical S-adenosyl methionine domain-containing protein 2; Interferon-inducible iron-sulfur (4FE-4S) cluster- binding antiviral protein which plays a major role in the cell antiviral state induced by type I and type II interferon. | 0.993 |
| IFI6 | Interferon alpha inducible protein 6 (138 aa) | 0.991 |
| IFI35 | Interferon-induced 35 kDa protein; Not yet known (288 aa) | 0.986 |
